# Fibril-Guided Three-Dimensional Assembly of Human Fibroblastic Reticular Cells

**DOI:** 10.1021/acsabm.4c00331

**Published:** 2024-05-28

**Authors:** Ketki Y. Velankar, Wen Liu, Paul R. Hartmeier, Samuel R. Veleke, Gayathri Aparnasai Reddy, Benjamin Clegg, Ellen S. Gawalt, Yong Fan, Wilson S. Meng

**Affiliations:** † Graduate School of Pharmaceutical Sciences, Duquesne University, Pittsburgh Pennsylvania 15282, United States; ‡ Allegheny Health Network Cancer Institute, Allegheny Health Network, Pittsburgh Pennsylvania 15212, United States; § Department of Chemistry and Biochemistry, Duquesne University, Pittsburgh, Pennsylvania 15282, United States; ∥ Department of Biomedical Engineering, Carnegie Mellon University, Pittsburgh,Pennsylvania 15213, United States; ⊥ McGowan Institute for Regenerative Medicine, University of Pittsburgh, Pittsburgh, Pennsylvania 15213, United States

**Keywords:** lymph node, lymph node stromal cells, self-assembling peptide, cell therapy, T cell tolerance, spheroids, 3D cell culture, injectable scaffold

## Abstract

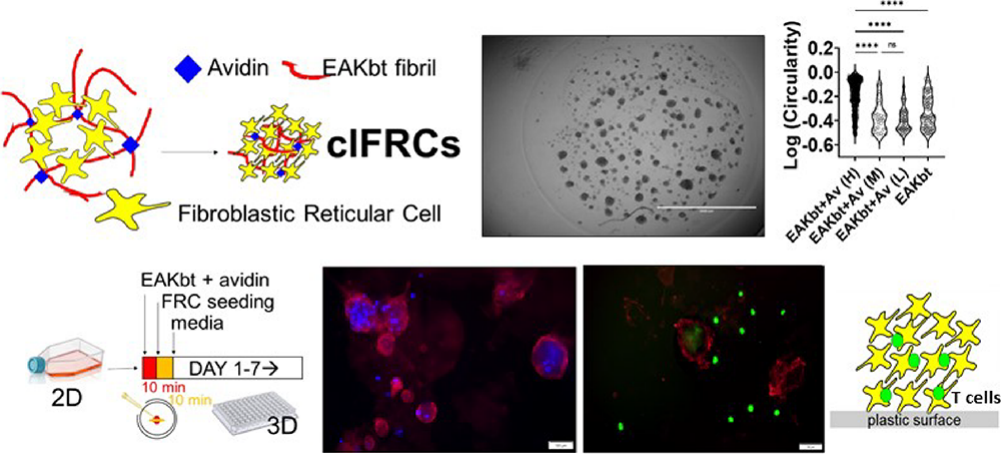

Fibroblastic reticular cells (FRCs) are stromal cells (SCs) that can be isolated from lymph node (LN) biopsies. Studies have shown that these nonhematopoietic cells have the capacity to shape and regulate adaptive immunity and can become a form of personalized cell therapy. Successful translational efforts, however, require the cells to be formulated as injectable units, with their native architecture preserved. The intrinsic reticular organization of FRCs, however, is lost in the monolayer cultures. Organizing FRCs into three-dimensional (3D) clusters would recapitulate their structural and functional attributes. Herein, we report a scaffolding method based on the self-assembling peptide (SAP) EAKII biotinylated at the N-terminus (EAKbt). Cross-linking with avidin transformed the EAKbt fibrils into a dense network of coacervates. The combined forces of fibrillization and bioaffinity interactions in the cross-linked EAKbt likely drove the cells into a cohesive 3D reticula. This facile method of generating clustered FRCs (clFRCs) can be completed within 10 days. In vitro clFRCs attracted the infiltration of T cells and rendered an immunosuppressive milieu in the cocultures. These results demonstrate the potential of clFRCs as a method for stromal cell delivery.

## 1 Introduction

Lymph nodes are hubs of antigen presenting cells and leukocytes that regulate the initial immune responses and maintain immune homeostasis.^1−4^ The reticular conduit in lymph nodes is organized around nonhematopoietic stromal cells, among which are fibroblastic reticular cells (FRCs). Broadly defined by their surface expression of GP38 and MHC-II molecules and the lack of CD45 and CD31 markers,^5^ FRCs render not only the structural cues for migrating leukocytes to adhere and interact, but also serve to steer immune responses and maintain peripheral tolerance.^6,7^ Within the lymph node stromal cell (LNSC) populations, multiple FRC subsets have been classified based on their approximate locations and phenotypes into marginal reticular cells, medullary FRCs cords, T/B border cells, and T zone FRCs in the paracortex region. FRCs in the T cell zone produce chemokines (e.g., CCL19, CCL21) to drive colocalization of dendritic cells (DCs) and T cells. Subsets of FRCs also produce IL-7, which is essential for T cell survival,^8^ and present peripheral tissue antigens (PTAs) via MHC class I and class II molecules through which autoreactive T cells are deleted and regulatory T cells (Tregs) are expanded.^9−13^

FRCs contribute to immunological tolerance through direct and indirect mechanisms. FRCs can directly induce Tregs through presentations of PTAs via MHC-II and PD-L1^2^ or indirectly by imprinting tolerogenic phenotypes into DCs.^14^ Blocking or deleting MHC-II in FRCs results in increased autoreactive T cells.^15^ FRCs impaired by chronic inflammation exhibit reduced expression of chemokines and cytokines, become senescent, and adopt a myofibroblast (fibrotic) phenotype,^16,17^ which can result in loss of T cell tolerance.

In vitro and in vivo evidence indicates that FRCs possess the capacity to inhibit the expansion of effector T cells during an immune response.^18^ In murine models of graft versus host disease (GVHD), FRCs have been found as hubs for the proliferation of Tregs,^19,20^ and de novo conversion of conventional T cells to Tregs.^14,19,21,22^ Acute GVHD damages the FRC network and reduces the presentation of PTAs, which is essential for deleting autoreactive T cells.^23^ In rodents, repetitive allograft transplantations drove FRCs to deposit excessive collagens,^24^ resulting in an altered lymph node microarchitecture coinciding with decreased Tregs and graft rejection. In mice transplanted with heart allografts, infusion of FRCs restores the capacity of CD40L blockade to induce long-term graft tolerance mediated by expansion of Tregs.^24^ Infusion of healthy FRCs into laminin α4-deficient mice restores tolerance to allografts.^23^ There is also direct evidence supporting the use of FRCs as cell therapies. Fletcher et al. showed that a single dose of mouse FRCs given intraperitoneally reduced mortality in mice undergoing sepsis.^25^ Transfer of FRCs restored normal architecture and reduced fibrosis in kidney draining lymph nodes damaged by infusion-related injury.^23^ While human FRCs can be isolated from routine lymph node biopsy, few studies have investigated the potential for delivering the cells as therapeutics. Within this context, we postulated that FRCs can be formulated into injectable multicellular spheroids.

A barrier in the delivery of the LNSC subset is that single-cell infusion of FRCs lose their reticular cohesion and consequently aspects of their functional attributes.^26^ Badillo et al. showed increased cellularity of FRCs seeded in 3D scaffolds relative to monolayer cultures.^27^ Another study showed that FRCs constructed in 3D spheroids enhanced antitumor immunity in mice.^28^ We have taken a bottom-up, self-assembling approach to formulate single-cell FRCs into three-dimensional (3D) clusters. A system of cross-linking fibrils was designed to guide the cohesion of human FRCs into 3D clusters. Using the self-assembling peptide (SAP) peptide AEAEAKAKAEAEAKAK (EAKII or “EAK” here) biotinylated at the N-terminus (EAKbt) cross-linked with avidin (EAKbt-av), the FRCs were congregated into multicellular units in the culture. Herein, we present data showing that clFRCs consist of viable GP38+ cells and have the capacity to retain and engage T cells.

## 2 Materials and Methods

### 2.1 Peptides, Proteins, and Other Reagents

Biotinylated EAK peptide (>95% purity) was synthesized and procured from Biomatik (Wilmington, DE). Avidin from egg (>99%) was procured from Millipore Sigma (Burlington, MA). Stock solutions of EAKbt (10 mg/mL) and avidin (5 mg/mL) were prepared by reconstitution with endotoxin-free sterile water and stored under frozen conditions until further use. Human lymphatic fibroblasts isolated from human donors and fibroblast complete growth medium were procured from ScienCell (Carlsbad, CA). Cells were cultured in poly lysine coated flasks at 37 °C and 5% CO_2_. Human peripheral blood mononuclear cells (PBMCs) were purchased from Charles River Laboratories (Wilmington, MA) and Lonza (Morristown, NJ). Fetal bovine serum was purchased from ATCC (Manassas, VA). Penicillin–streptomycin, Actin dye, Congo red, l-glutamine, Dulbecco’s phosphate-buffered saline (DPBS), and endotoxin-free sterile water were purchased from ThermoFisher Scientific (Waltham, MA). The antihuman GP38 primary antibody and isotype antibody were purchased from Abcam (Waltham, MA). The Alexa-Flour 555 antirabbit secondary antibody was purchased from BioLegend (San Diego, CA).

### 2.2 Formulation of EAKbt-Avidin Assembly and FRC 3D Cell Clusters

**Figure 1:**
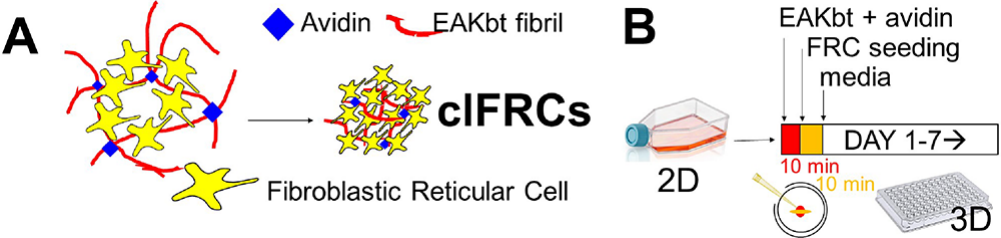
Facile and scalable method is used to generate 3D clustered FRCs (clFRCs). (A) Assembly of single-cell FRCs into 3D clusters through combined forces of fibrillization and biotin–avidin interactions; (B) FRCs propagated from primary cells as a monolayer are seeded in 96-well tissue culture-treated microtiter plates at 2.5 × 10^4^ cells into 7 μL of EAKbt-av followed by adding complete media to a total of 200 μL per well and cultured up to 7 days.

A peptidic assembly composed of cross-linked EAKbt for generating 3D FRC clusters was formulated by mixing EAKbt and avidin in predetermined ratios (Figure 1). Briefly, avidin was added to EAKbt in a cell culture-treated plate at varying molar ratios of EAKbt:avidin at 29:1, 144:1, and 1437.5:1. The materials were allowed to incubate for 10 min at room temperature to allow cross-linking between EAKbt and avidin. After the formation of the biomaterial scaffold, CD45-GP38+ CD31- human lymphatic fibroblasts (HLFs) were seeded at a concentration of 0.25 × 10^5^ cells (5 μL) per well within the assembly. After incubation for 10 min at room temperature, 200 μL of fibroblast complete medium was added to each well. Cells cultured as monolayers in flasks between passages 7 and 15 from the vial received were used to generate the clusters. The cells were supplemented with fibroblast complete medium and cultured for predetermined durations at 37 °C and 5% CO_2_.

### 2.3 Characterization of EAKbt-Avidin Peptidic Assembly

The EAKbt-av assembly was formed by mixing EAKbt and avidin at predetermined molar concentrations in 96-well plates, as described above. The final volumes of the cross-linked EAKbt and the controls were maintained constant. The assembly was allowed to form by avidin-mediated cross-linking followed by sample pretreatment for the respective characterization techniques. EAKbt-av samples were analyzed using a scanning electron microscope (SEM) to reveal the scaffold structure at high resolutions. The samples were prepared on the carbon dot mount, as described above. The samples were air-dried and the images were captured on a Zeiss Sigma VP SEM (Oberkochen, Germany) with at 1 kV accelerating voltage.

The formation of EAK β-sheet fibrillar assemblies was tested by studying the UV absorbance using Congo red (ThermoFisher), which exhibits a spectral shift in the presence of β-sheet structures. Congo red was incorporated in the EAKbt assembly by the addition of a PBS diluted stock solution to the cross-linked or self-assembled EAK. The shift in the UV absorbance spectra was determined by reading the absorbance of the samples using a TECAN infinite M1000 microplate reader (Männedorf, Switzerland). The Congo red-stained scaffolds were visualized using an IX 73 epi-fluorescent inverted microscope (Olympus, Center Valley, PA). The pore size determination was performed using cellSens software (Olympus). The captured images were subjected to thresholding followed by calculation of the pore area.

The porosity of the clusters was determined based on the retention of dextran molecules of different molecular weights, 10 and 70 kDa, labeled with Rhodamine B (Invitrogen). Preformed clFRCs were incubated with dextran solutions overnight and washed five times with phosphate buffered saline (PBS) to remove unbound dextran. Retention was determined based on imaging using an Olympus IX 73 epi-fluorescent inverted microscope.

### 2.4 Formation of FRC Clusters Using Alternate Surfaces and Stress Testing for Injectability

To evaluate if the cluster formation is a surface-dependent phenomenon, FRCs were cultured in Eppendorf tubes (low protein binding, non-cell culture treated plastic) following the same method as described above. The translational feasibility of the formed clusters to preclinical and clinical models was further investigated. The formulated clusters were subjected to stress tests that would be encountered while injecting the formulation by passing through a 25^1/2^ gauge syringe needle a total of 20 times. The FRC clusters were collected, and morphological features were determined as described below.

### 2.5 Morphological Characterization of Generated FRC Cell Clusters

The cell clusters formed using EAKbt-av system were visualized using a bright-field microscope (EVOS imaging systems, ThermoFisher). The morphological features of individual clusters were quantified by analyzing the images using Fiji/ImageJ software (NIH, Bethesda, MD). Morphological features for each FRC cluster were quantified from processed images by using built-in analysis algorithms. Briefly, each image was processed through a threshold to enhance the contrast. The clFRC clusters were identified from the threshold adjusted image using the “analyze particles” plugin by setting the lower limits of area (1000 μm^2^) and circularity (0.3) to eliminate background noise. Circularity was used as one of the parameters indicative of the 3D nature of clusters from the captured 2D bright-field images. The circularity of clusters was determined using the following equation:
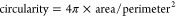


Additional cluster readouts such as cluster numbers per well and cluster area and aspect ratio were determined by following a similar image analysis methodology. Changes in cluster features as a function of culture duration and the effect of varying avidin concentrations were studied by processing images captured from the respective culture conditions.

### 2.6 Organization of FRCs within the Clusters and Intercluster Interactions

Organization of FRCs within the biomaterial scaffold was evaluated by staining the FRCs using actin staining dye (Invitrogen) as per manufacturer’s protocol. Briefly, cells were fixed using 4% formaldehyde for 60 min and incubated with the actin dye (1:2000 diluted in DPBS) for 60 min at room temperature. The biomaterials were stained using Congo red, as previously described. The nuclei of the cells were stained using a 1:2000 Hoechst dye (ThermoFisher) and used to determine the cell number per cluster. Images were captured using an IX 73 epi-fluorescent inverted microscope (Olympus, Center Valley, PA). The captured images were analyzed by using ImageJ software to identify cell clusters and quantify the fluorescence signal intensity area occupied by each cluster. The % void area was calculated as
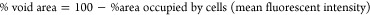


Further organizational insights into the cellular assembly of 3D FRCs in response to EAKbt+Av were obtained by a confocal microscope (Nikon, Japan). Images were captured as z stacks with a set step distance of 5 μm, and 3D construction was conducted using the NIS-element software (Nikon).

### 2.7 Expression of Hallmark FRC Markers on 3D Clusters by Immunofluorescence

The formulated FRC clusters were studied for the expression of podoplanin (GP38) which is a characteristic marker of FRCs. GP38 expression was studied by incubating the FRC clusters with an anti-GP38 primary Ab (Abcam) for 60 min followed by incubation with dye-labeled secondary Ab. Control groups included samples treated with an isotype antibody or secondary antibody without the primary; EAKbt-av without FRCs undergoing the same processing methodology were employed for determining any nonspecific signals. All images were captured using an IX 73 epi-fluorescent inverted microscope (Olympus, Center Valley, PA). Fluorescent images were quantified for their mean signal intensity by using ImageJ software.

### 2.8 Immune cell retention in FRC clusters

The human lymphoma T cell line (Jurkat) was stained using 1 mM CFSE (carboxyfluorescein diacetate succinimidyl ester) dye as per manufacturer’s protocol. CFSE-labeled Jurkat cells were added to 96 well plates containing FRC clusters cultured until day 7 and stained for GP38 as described above. Jurkat cells were allowed to incubate for 2 h with the FRCs followed by harvesting and washing the FRC clusters with 200 μL of DPBS for a total of 5 times to remove the nonentrapped Jurkat cells. The wells were supplemented with 100 μL of fresh complete medium, and images were captured using an Olympus microscope and cultured further until 7 days. The retention of human PBMCs (Lonza) was tested by staining the cells with CFSE, activating with aCD3/aCD28 activator, and coculturing for 5 days. The PBMC and FRC clusters were washed with DPBS 5 times as described above, and images were captured using a Olympus IX 73 epi-fluorescent inverted microscope.

### 2.9 Live/Dead Assay

The viability of the FRCs in the presence of EAKbt, EAKbt + avidin, and monolayer were analyzed using a live/fead staining kit (Fisher) as per manufacturer’s protocol. Briefly, the live cell (green) and dead cell dye (red) were mixed prior to addition. 20 μL of the dye mixture was added to each well and incubated for 10 min. Cells were subjected to multiple washes with 200 μL of DPBS and the images, were captured using the Olympus fluorescent microscope.

### 2.10 Coculture of 3D FRC Clusters with PBMCs from Human Donors

The biological immunoregulatory effect of the FRC formulation on immune cells was evaluated by coculture with human PBMCs. FRC clusters were formulated in 96 well plates as described above for 7 days, and each well was added with 1.3 × 10^5^ PBMCs and allowed to further culture for 7 days. For studies with activated PBMCs, the cells were activated using an Immunocult CD3/CD28 T cell activator (STEMCELL Technologies, Cambridge, MA) before addition to the coculture in complete medium supplemented with 10 IU/mL of recombinant human IL-2. Each test was performed in replicates with additional replicates with independent FRC passages and frozen stocks.

### 2.11 RT-PCR

PBMCs from coculture experiments were separated from the clFRCs followed by separate RNA extraction from the PBMC and the clFRC fraction. RNA was extracted by using the RNeasy Mini kit (Qiagen). RNA was reverse transcribed by using the Superscript III First Strand Synthesis System (Invitrogen). Real-time qRT-PCR was performed on Roche LightCycler 480 II using All-in-one qPCR Mix (Genecopoeia) for glyceraldehyde-3-phosphate dehydrogenase (*GAPDH*, endogenous control, assay ID Hs02758991_g1) and FRC-related genes for the FRC fraction of coculture and regulatory T cell genes for the PBMC fraction. Data from each gene of interest for each sample were normalized to the expression of *GAPDH*. These data were then expressed as relative expression to the housekeeping gene or as fold-change relative to the level of untreated PBMCs using the delta Ct method.

### 2.12 Statistical Analysis

All statistical analyses were performed using the GraphPad Prism 9.0 software (GraphPad, San Diego, CA). The statistical significance between different experimental groups was assessed using a paired or unpaired *t* test (for comparing two groups) or by a one-way ANOVA with multiple comparisons. For data sets not following a normal distribution, log transformation of data was performed prior to the application of parametric tests for statistical significance. All data sets in graphs are represented as mean ± standard error of the mean. The *p* values are indicated by the asterisks where * represents *p* < 0.05, ** *p* < 0.01, *** *p* < 0.005, and **** *p* < 0.001 while “n.s.” indicates nonsignificant.

## 3 Results

We sought to construct a multicellular compact of FRCs within which T cells could be retained and regulated. The premise was that congregated FRCs would generate gradients of chemokines for attracting and colocalizing T cells and DCs. Assemblies of FRCs were created by intermixing the cells suspended as single cells with SAPs cross-linked by biotin and avidin. The cells and biomaterials coalesced into clusters consisting of viable cells exhibiting the phenotypic attributes of the LNSC subset.

### 3.1 Avidin Cross-Linked EAKbt Forms a Fibrillar Reticular Matrix

**Figure 2:**
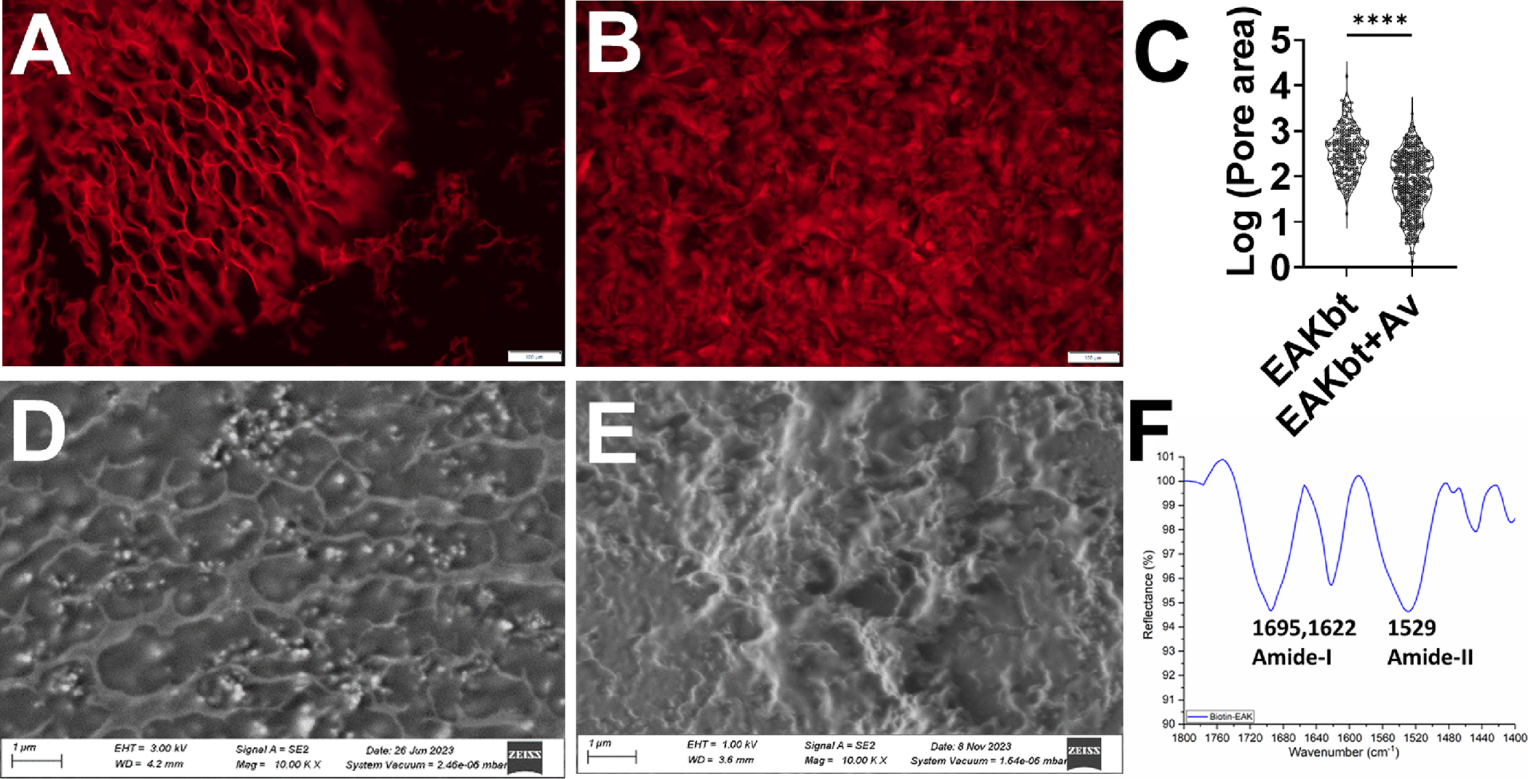
Reticular fibrillar network in the EAKbt-av scaffold. Congo red illumination of (A) EAKbt and (B) EAKbt-av, which shows a dense porous network formed by avidin cross-linking [scale bar = 100 μm]; (C) avidin reduces the mean pore area in EAKbt (11.29 μm ± 0.37 vs 23.93 μm ± 1.09) SEM micrograph showing the (D) EAKbt and (E) EAKbt-av fibril network; (F) FTIR analysis of EAKbt showing peaks corresponding to beta-sheet structure. In addition, the formation of β-sheets in EAKbt was supported by UV absorbance analysis showing a shift of 14 nm in the maximum absorbance of Congo red in saline at (484 nm); the absorbance shift was retained in EAKbt+av (shift = 14 nm) and corresponded with the parent EAK absorbance shift (18 nm).

We first characterized the physical features of SAP EAKbt using microscopic and spectroscopic methods to understand the impact of the cross-linking. The avidin–biotin interaction, with *K*_*D*_ reported in the range of 10^–14^ to 10^–15^ M,^29,30^ drives a spontaneous and irreversible complexation on practical time scales.^31^ As expected, EAKbt self-assembles into β-sheet-rich structures resembling the fibrils of the parent SAP EAK (Figure 2 A,B,D,E). Infrared spectroscopy shows EAKbt registering distinct Amide I and Amide II peaks, which are signatures of higher-order β-sheet structures (Figure 2F). The fibrillization of EAKbt was confirmed based on the UV absorption peak of Congo red undergoing a red shift that is characteristic of the dye binding to β-sheet structures^32^; both EAKbt and EAKbt+av increased the λ_max_ from 484 to 504 nm. These results indicate the preservation of the EAKbt β-fibrils in the presence of avidin.

Formation of a scaffold assembly was observed in fluorescent microscopic images that revealed EAKbt forming a mesh-like scaffold comprising interwoven fibrils (Figure 2A) with an average pore size (diameter) of 23.93 μm ± 1.09 (standard error). Intermixing EAKbt-av resulted in denser assemblies of compact fibrils (Figure 2B). On the other hand, an average pore size of 11.29 μm ± 0.37 was measured in EAKbt-av, a system of soft materials in which immune cells could infiltrate (Figure 2C). SEM images confirmed that adding avidin transformed the EAKbt fibrils to densely packed 3D structures (Figure 2D,E). These results indicate that avidin transforms EAKbt into a reticular fibrillar composite of porous networks.

### 3.2 EAKbt-Avidin Induces Assembly of FRCs into 3D Clusters

**Figure 3:**
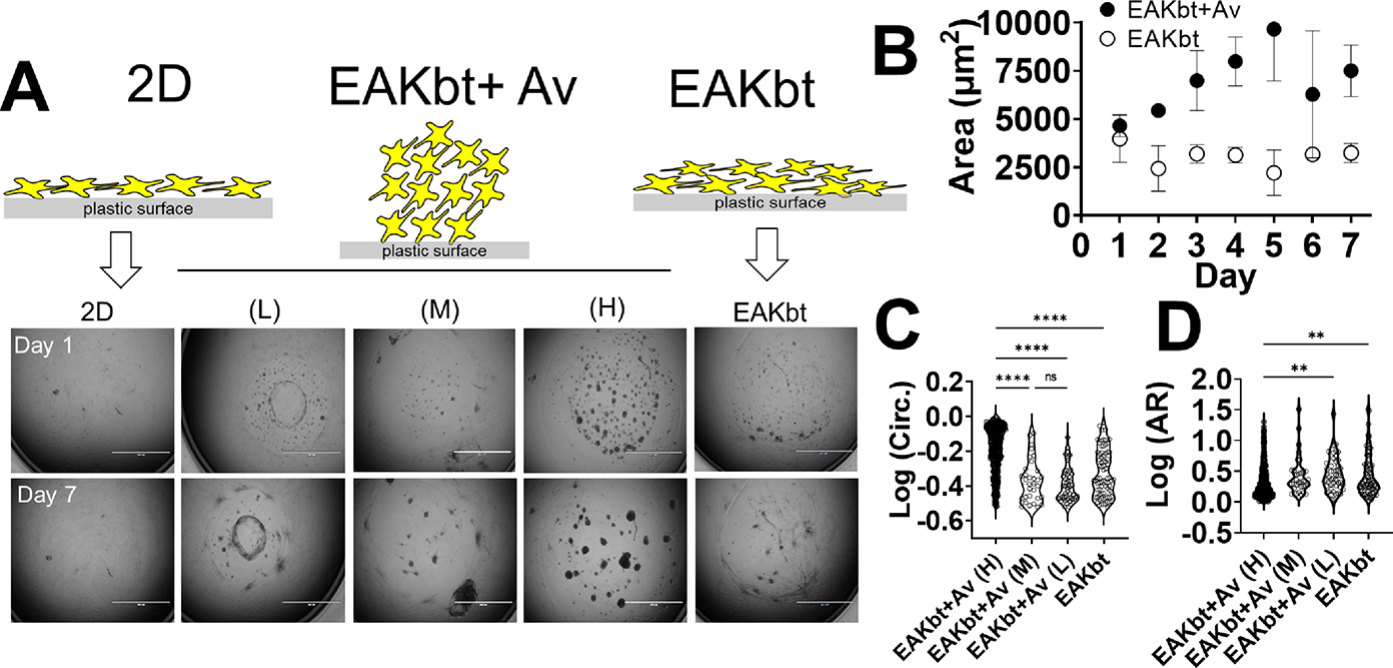
Generation of stable FRC clusters in EAKbt-av. (A) Clusters formed as a function of avidin concentration; no cluster was observed in monolayer and EAKbt induced only surface-adhered aggregates; clusters progressively emerged over time from d1 to d7 [scale = 2000 μm]; (B) mean cluster size increased over 7 d in culture without additional manipulation; 3D nature determining parameters correlated with the degree of cross-linking as indicated by the morphological parameters, namely, (C) circularity and (D) aspect ratio (AR); the analyses were performed in ImageJ/Fiji with BF TIFF images converted into binary images and subjected to detection of particles with area >1000 μm^2^ and circularity of at least 0.3; H, M, and L represent formulations with varying molar ratios of EAKbt: avidin at 29:1, 144:1, and 1437:1, respectively.

We speculated that the physical architecture of EAKbt-av is conducive for encapsulating FRCs into a functional compact. Conceptually the cross-linking fibrils create proximal 3D anchoring points, thereby driving the cells into reticular cohesion at the expense of surface adhesion. To test this supposition, FRCs were harvested from human lymph nodes, maintained as monolayer cultures, and resuspended as single cells immediately prior to seeding with EAKbt and avidin (Figure 1). The assembly was induced by admixing the cell suspension into EAKbt and avidin spotted in the center of a microtiter plate, tissue culture-treated (TC) well (Figure 1B). TC plates were used to determine the capacity of the biomaterials to compete with surface adsorption for the anchoring of FRCs. The suspended FRCs were seeded into EAKbt and avidin mixed at different molar ratios (Figure 3). At the indicated time points, the cultures were imaged under bright field (BF) microscopy at 2× magnification at which the entire field could be captured in a single image in order to allow for global and detailed analyses. The captured images were analyzed by using ImageJ/Fiji to quantify the number and size of the clusters and other morphological features.

**Figure 4:**
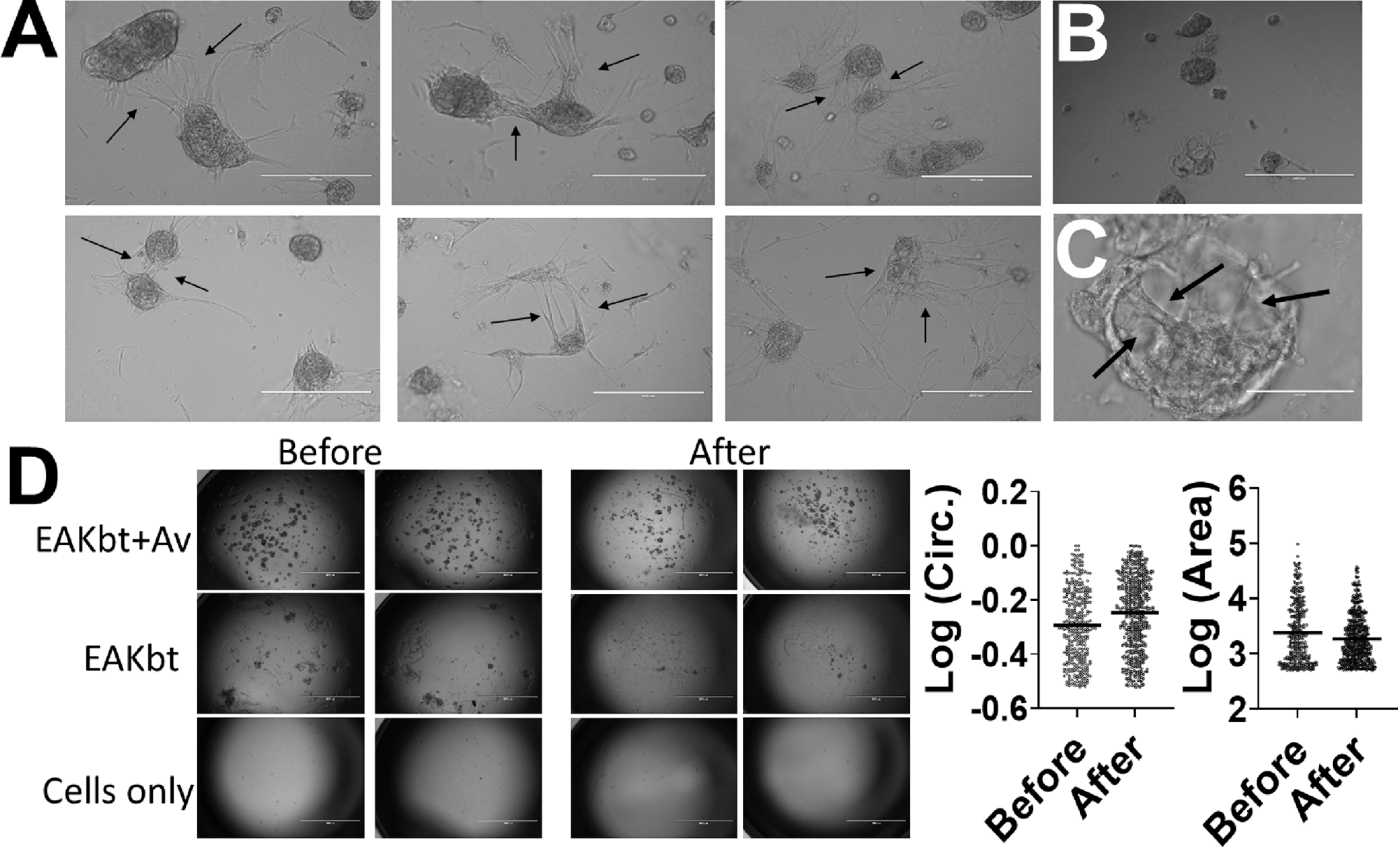
Intercluster cellular connectivity and injectability of FRC clusters. (A) Linked protrusions between clusters [scale = 400 μm]; (B) clusters formed in Eppendorf tubes, indicating the cluster formation as a function of the EAKbt+av and not a surface driven effect [scale = 400 μm]; (C) cellular connections within a FRC cluster highlighting the 3D reticular organization of FRCs [scale = 100 μm]; (D) clusters sustained syringe shear stress (25^1/2^G needle); morphological characterization of clusters before and after syringe shear test [scale = 2000 μm]. Binary images were subjected to detection of particles with an area above 500 μm^2^ and circularity of at least 0.3.

As expected, wells containing FRCs without the biomaterials spread into monolayers, and no clusters were found during the 7-day period (Figure 3A). Cells seeded in EAKbt without avidin were found to mostly adhere to the surface, although a few multicellular aggregates could be seen (Figure 3A). The combination of EAKbt and avidin (EAKbt+av) drove almost all the cells into clusters. The effect of the cross-linking on the cells was further evidenced in wells containing EAKbt mixed with avidin at three different molar ratios. The number of clusters increased as the concentrations of avidin increased, suggesting the assembly process was a function of the degree of the cross-linking. The clusters generally adopted a circular morphology, with an average diameter of 138.3 μm ± 3.66 (standard error). The effect of the cross-linking was more notable after 7 days in culture, with significantly larger clusters found in samples containing higher concentrations of avidin (Figure 3A,B). Intriguingly, some of the clusters appeared to form connecting protrusions (Figure 4A), a feature analogous to the tunneling nanotubes observed in DCs, neuronal cells, and mesenchymal stem cells (MSCs).^33^ These results support the notion that the cross-linking EAKbt fibrils guided the cells to 3D clusters.

The clusters were further characterized for their morphometric attributes using ImageJ/Fiji. The BF images were transformed into binary data, with the threshold adjusted to enhance the contrast. Clusters were identified using the “Find Particles” function. For each of the clusters, different features including circularity, aspect ratio, and Feret diameter were calculated. These metrics were used for determining the apparent 3D nature of clusters on the basis that images captured from the top view will show morphologies distinct from the elongated shapes of cells spread as a 2D monolayer. As adherent cells, FRCs attached to the tissue culture-treated surface in 2D monolayer and display spindle-like shape and appear elongated. In contrast, a 3D assembly of FRCs would constitute nonsurface attached cells thereby appearing less elongated and more circular. Therefore, the image analysis enables the differentiation of 3D vs 2D nature based on the calculated circularity value, with a near-perfect circle having a value approaching 1. For the current purpose, we defined monolayer or stacked cells as having circularity values <0.5. Collective analysis of multiple wells across multiple plates and independent cultures showed that the EAKbt-av clustered FRCs (clFRCs) mostly consisted of 3D clusters, while adherent or aggregates of 4–5 cells were found in the FRCs formulated with EAKbt without avidin. These observations were confirmed in circularity and aspect ratio (Figure 3C,D), with both indicating that the majority of the clusters were compact in shape. Taken together, these features support the notion that clFRCs are 3D multicellular units and that cross-linking is a critical factor in driving cell assembly. Because the clusters with the highest avidin concentration were formed more consistently and rapidly, the EAKbt-av (H) formulation was used in the subsequent experiments.

### 3.3 Surface Independence and Injectability of the FRC Clusters

We next determined the extent to which clustering could occur on a different surface. Instead of spotting EAKbt+av in wells of microtiter tissue culture-treated plates, the biomaterials were deposited at the tip of a “low protein binding” microcentrifuge tube before intermixing with FRCs suspended as single-cell. Complete media (200 μL) were then added immediately to the tube to begin the 7 day incubation. The same number and density of FRCs were deposited into the same volume and concentrations of EAKbt and avidin as those prepared in the microtiter plate method. BF images show that similar to the microplate-based method, EAKbt+av drove the formation of clusters in tubes (Figure 4B). The clusters yielded a similar circularity score, while no clusters were found in the microcentrifuge tubes deposited with FRCs without the biomaterials. Figure 4C shows a representative image highlighting the arrangement of FRCs within a cluster. Thus, the clustering of FRCs in EAKbt+av was therefore determined as independent of the surface; the ability to generate clFRCs in suspension is conducive to scaling up production.

Analogous to the delivery of MSC^34−37^ and thymic epithelial cells (TEC),^38−41^ injectability is another prerequisite for ectopic cell therapies.^42,43^ Similar to MSC spheroids, clFRCs must sustain the shear stress exerted during syringe injection. To this end, we tested the integrity of clFRCs by passaging the clusters repeatedly (10 times) through a syringe fitted with a 25^1/2^G needle. While cells mixed with EAKbt suffered a significant loss in assemblies (based on the area occupied), clFRCs (i.e., FRCs in EAKbt+av) retained most of the starting clusters (Figure 4D). The slight increase in the circularity of clFRCs after being subjected to the stress test was likely due to shear-induced disintegration of loosely associated FRCs (Figure 4D). Taken together, these results indicate that the cell–cell and cell–fibril interactions in the FRCs clusters could sustain the shear stress in conventional needle syringe injection. Taken together, these results direct the potential of clFRCs to sustain the injection forces for *in vivo* administration.

### 3.4 clFRCs Are Compact Spheroids Containing Viable GP38+ cells

**Figure 5:**
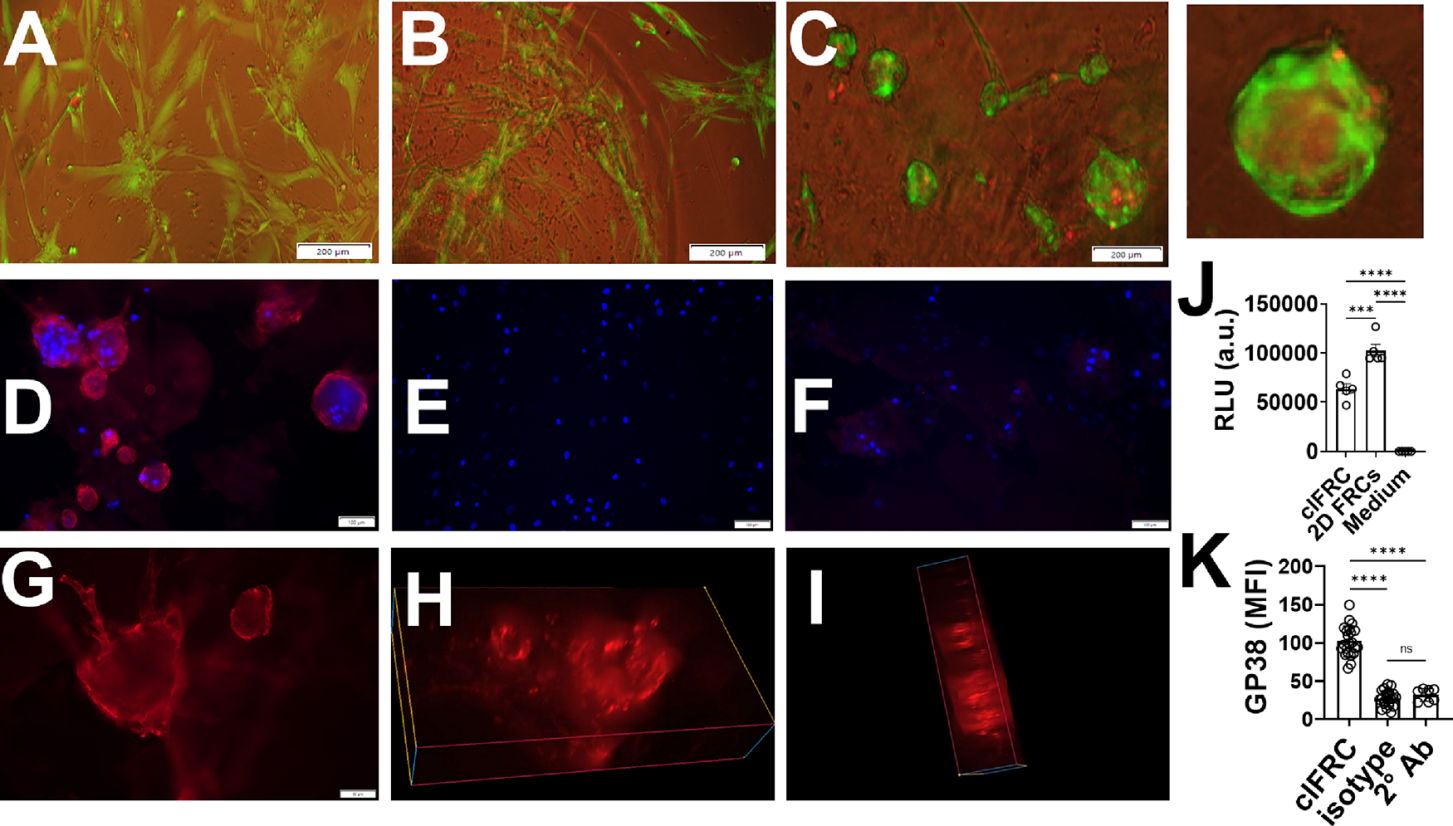
Biological attributes of FRC clusters in EAKbt-av. Live/dead staining of FRCs in (A) 2D monolayer, (B) EAKbt, and (C) EAKbt+av wherein viable cells are observed in clusters [scale = 200 μm]. Immunofluorescent (IF) images with FRCs stained with GP38 (red) and DAPI (blue) [scale = 100 μm] (D) clFRCs, (E) isotype antibody control, (F) 2°Ab control; (G) 3D GP38 slice in clFRCs [scale = 50 μm]; (H&I) image reconstruction from Z-stack IF images demonstrating a spherical 3D assembly of FRCs in clusters (EAKbt+av); (J) ATP assay showing presence of metabolically active FRCs; and (K) GP38 relative expression to isotype and 2°Ab controls in immunofluorescent (IF) images.

The multicellular organization in clFRCs was further analyzed by using immunofluorescence (IF). We found that the typical clusters encapsulated live cells but also a necrotic core (Figure 5C), which is consistent with the notion that the FRCs undergo constant turnover, with the exterior containing newly proliferated cells, pushing aged and dead cells into the interior. To confirm the potential of FRCs to maintain a turnover of cells in the clusters, clFRCs were analyzed for metabolically active cells using an ATP-based assay. clFRCs were found to be metabolically active, thus suggesting the potential to replenish the live cells within clFRCs (Figure 5J). Next, we investigated the extent to which clFRCs retained their characteristic biomarkers in the 3D form. The clustered FRCs were found to express GP38, which is expressed by the LNSC subset, with each containing up to 25–30 number of cells in a given plane of DAPI-stained nuclei (Figure 5D–F). The expression of GP38 was confirmed based on quantifying the mean fluorescent intensities (MFI) relative to staining with an isotype control antibody (Figure 5K). These results show that the typical cluster contains at least several dozen viable GP38+ cells.

**Figure 6:**
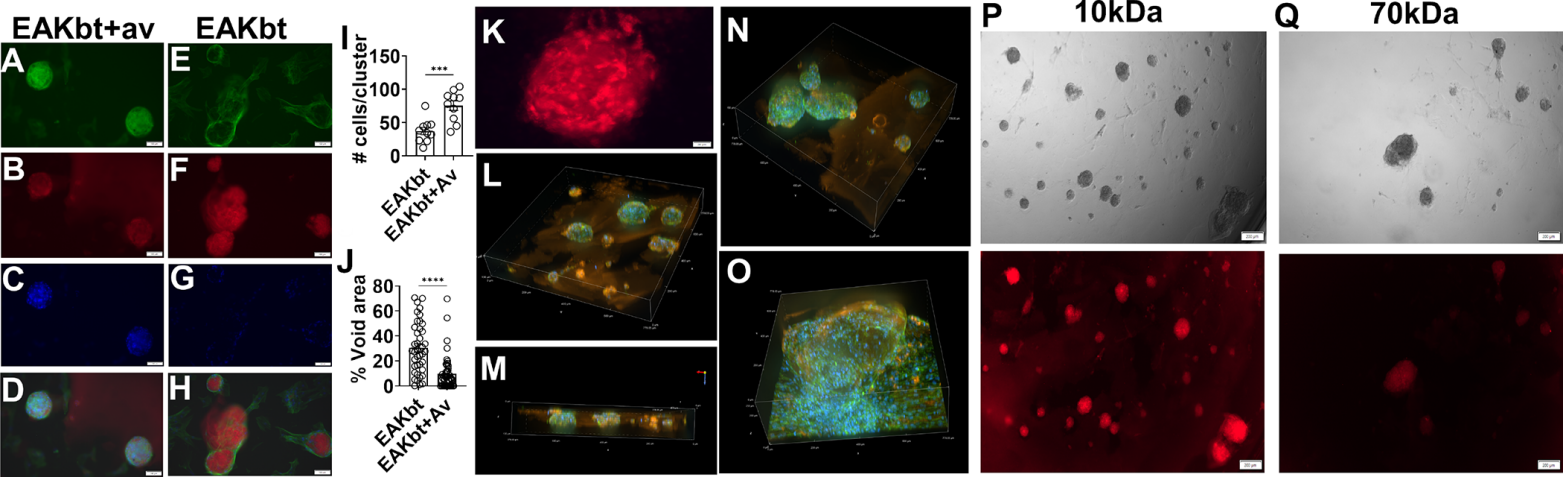
Effect of EAKbt+av on the organization of FRCs in clusters. The positive effect of avidin cross-linking was demonstrated in contrasting FRCs assembled in the presence (A–D) and absence (E–H) of avidin stained with actin (green), outlining the cytoskeleton, SAP fibril (red), nuclei (blue), and in the overlay [scale = 100 μm]; (I) cell#/cluster based on DAPI; (J) area for cell occupation relative to EAKbt; (K) Congo red stained clFRCs [scale = 20 μm] revealing the porous nature of the EAKbt+av assembly supporting clFRCs, 3D view constructions of the confocal microscopic imaging of (L,M,N) clFRCs and (O) EAkbt stained with actin (green), outlining the cytoskeleton, EAKbt (orange), nuclei (blue); 3D image reconstruction confirms the spheroid nature of cells in the clFRCs; Microscopic images [scale = 200 μm] showing retention of fluorescent labeled dextran in clFRCs when tested with (P) 10 kDa and (Q) 70 kDa dextran grades. Statistical analysis was performed using a two tailed unpaired *t* test where *** and **** indicates *p* < 0.001 and *p* < 0.0001, respectively.

The 3D nature of the clFRCs was also evident in multifocal microscopic imaging (Figure 5G–I). Co-localization of FRCs (actin stained) and SAP fibrils (Congo red) shows that without avidin, EAKbt folded into scaffolds, but the fibrillar structures generally appear “hollow”, with cells mostly organizing around the exterior (Figure 6E–H). In contrast, clFRCs (EAKbt-av) exhibit a solid core, consistent with the notion that the 3D volume was mostly occupied by the cells (Figure 6A–D); the cell density estimated (based on stained nuclei) was significantly higher in clFRCs than in FRCs admixed with EAKbt (Figure 6I). Additionally, clFRCs appear as a highly compact structure, with a lower % of void area relative to FRCs mixed with EAKbt (Figure 6J). The fine structure of the fibrils in clFRCs further supports a porous spheroids (Figure 6K). Confocal microscopic images captured at different z stack positions confirmed the dense assembly of FRCs in each cluster as described above (Figure 6L,N). The spherical nature of the clusters was evidenced by the 3D reconstruction of clusters with actin, nuclei, and SAP illuminated with their respective dyes (Figure 6M). The average diameter of several major clusters was measured to be ∼130 μm, which is consistent with the measurements made using the BF images (Figure 3B). Remarkably, all of the cells appeared localized inside the biomaterial clusters (Figure 6L–N), suggesting strong affinity of the cells for the avidin cross-linked SAP fibrils. In contrast, a confocal image of FRCs with only EAKbt without avidin shows a flattened multilayered assembly with cells inside and outside the biomaterials (Figure 6O). These observations highlight the critical role of cross-linking in compacting viable GP38+ FRCs into spheroids. Porosity of the clusters was further evaluated by testing entrapment of dye-labeled dextran of two molecular weights-10 kDa which approximidated ∼1 nm in molecular diameter and 70 kDa grade of ∼8 nm. Both species were found to be retained in the clFRCs (Figure 6P,Q).

### 3.5 clFRCs Retain T Cells and Drive an Immunosuppressive Milieu

**Figure 7:**
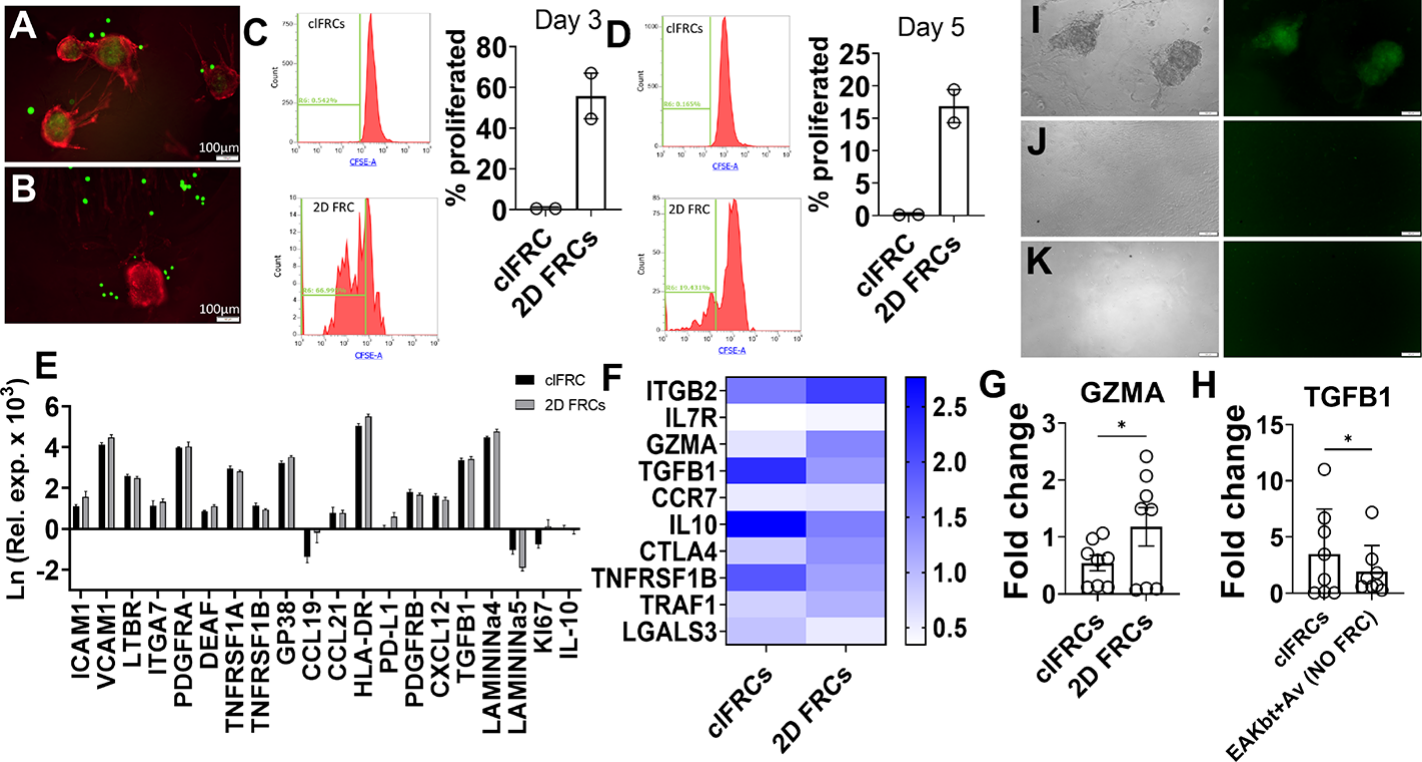
clFRCs retain and engage T cells. Superior retention of human T cell (green-CFSE labeled) in (A) clFRCs (red-GP38) relative to (B) EAKbt-FRCs [scale = 100 μm]; flow cytometric analysis of proliferation of CFSE labeled Jurkat cells at different culture durations of (C) day 3 and (D) day 5; (E–H) RT-qPCR of day 7 clFRCs/allo-PBMCs cocultures; (E) relative exp (GAPDH) of signature FRC genes in the clFRCs fraction; (F) relative exp of tolerance-inducing T cell genes in the lymphocytes fraction recovered from cocultures of clFRCs vs 2D monolayer. (G) Relative expression of Granzyme A (GZMA) in the lymphocytes fraction recovered from cocultures of clFRCs vs 2D monolayer; (H) relative expression of TGFB1 in the lymphocytes fraction recovered from cocultures of clFRCs vs biomaterial control (EAKbt+Av, no FRCs), CFSE-stained aCD3/aCD28 activated human PBMCs (green) cocultured with (I) clFRCs, wherein the PBMCs show infiltration and retention inside the FRC network, (J) 2D monolayer, and (K) EAkbt+Av (no FRC) [scale = 100 μm]. The results were generated from 4 to 5 technical replicates using FRCs from one donor and PBMCs from two donors. Statistical analysis was performed using a one-tailed paired *t* test where * indicates *p* < 0.05.

In the lymph node paracortical region, DCs, T cells, and FRCs congregate into a mutually supportive hub, in which peripheral tolerance is generated. The FRCs contributing to the reticular architecture provide the cues for the cells to adhere and communicate.^44−46^ In clFRCs, we investigated if the FRCs networks can interact with immune cells. We observed that T cells could invade the clFRCs. Jurkat cells labeled with the membrane dye CFSE added to clFRCs were found inside the clusters (Figure 7A), while neither EAKbt (without avidin) (Figure 7B) nor monolayer (2D) FRCs retained the T cells. We next sought to understand the biological effects of the clFRCs on the cocultured lymphocytes. CFSE stained Jurkat cells recovered from clFRCs cultures showed reduced proliferation relative to coculturing with 2D FRCs (Figure 7C,D), pointing to the potential suppressive nature of the 3D clusters. Studies have shown that FRCs secrete nitric oxide (NO), which inhibits expansion of effector T cells during an immune response.^18^ The microscopic images obtained after day 3 of coculture with PBMCs (Figure S2) show what appear to be lymphocytes congregating around the FRC clusters. These lymphocyte-cluster interactions were absent in the biomaterial only (no FRCs) group, suggesting that the organization is a function of FRCs. Closer examination revealed a general phenomenon in which the lymphocytes outlined the FRCs along the edges of the clusters. We also cocultured PBMCs stained with CFSE and activated with aCD3/aCD28 in the presence of FRCs. After 5 days of coculturing, the PBMCs were harvested, and the clusters were washed 5 times to remove unbound cells. The results indicate that clFRCs (Figure 7I) but not the control groups (Figure 7J, K) colocalized with PBMCs, suggesting the 3D clusters have the capacity to retain and interact with immune cells.

The immunological impacts exerted by clFRCs were examined in coculturing allogeneic human PBMCs with clFRCs for 7 days. At the end of the period, the adhered clusters (clFRCs) and suspended cells (PBMCs) were separated from the clusters and analyzed by using RT-qPCR. Cells in the adherent fraction expressed the signature FRC genes, including *GP38*, *PDGFRA*, *DEAF*, *CCL21*, and *CXCL12* (Figure 7E). Notably, laminin α4 was found highly elevated relative to α5, which is correlated with T cell tolerance.^16^ The suspended cells recovered from the cocultures also revealed an immunosuppressive phenotype, as evidenced in upregulated *IL-10*, *TGFB1*, and *TNFR2* (Figure 7F), which is expressed in a highly suppressive subset of Tregs.^47−50^ Moreover, a significant downregulation of Granzyme A (*GZMA*) was observed in the clFRCs fraction relative to that of 2D FRCs (Figure 7G). GZMA is upregulated in CD4 helper T cells in allogenic activations and in GVHD.^51^ When compared with the biomaterial control, clFRCs show an upregulation of TGFB1 (Figure 7H), a cytokine that drives the differentiation of regulatory T cells. Taken together, these results suggest clFRCs are functionally responsive.

## 4 Discussion

The results indicate that the combination of fibrillization and bioaffinity avidin cross-linked EAKbt drove the FRCs into the 3D structures observed in the clFRCs. The spherical nature of the clusters was established based on images taken in bright field, fluorescent, and confocal modes. Furthermore, the porous cell-biomaterial network permits the transport of nutrients and antigens and infiltration of lymphocytes and DCs. The clustered FRCs would render effective local gradients of chemokines/cytokines for attracting and retaining T cells as a step toward differentiation or polarization. Culturing cells under 3D conditions is a proven strategy for recapitulation of endogenous physiological niches. Conventional methods such as nonadhesive surface forced aggregation and hanging drop techniques generally lack the molecular cues provided by matrix-assisted anchoring.^52,53^

FRCs have been investigated as cell therapies in cancer and autoimmune diseases.^54^ While FRCs can be isolated, enriched, and expanded from lymph nodes through routine biopsies, the prospects of their clinical translation depend on the ability to administer these cells in vivo as cooperative functional units. However, few attempts have been reported to construct 3D spheroids of FRCs as the mode of delivery.^5,55^ The functional attributes of lymph node FRCs reside in part in their cohesive network organization. The physical cues presented in the natural porous collagenous matrices are necessary for the cells’ survival and responsiveness. This paradigm has been employed previously in bioaffinity EAK hydrogels developed for constructing organoids of thymic epithelial cells (TECs)^56^ and other tissues.^57,58^ We have shown that Fc-binding SAPs guided medullary TECs into injectable and functional multicellular units.^38−41^ The data shown here demonstrate the ability of a new bioaffinity EAK system in which the combined forces of biotin–avidin interaction and SAP fibrillization were leveraged in the assembly of human FRCs into viable, injectable, metabolically, and immunologically active spherical clusters.

The cell delivery formulation consists of avidin cross-linked EAKbt, a biotinylated SAP. The clustering was reproducible and robust as observed across independent cultures and HLFs lots (Figure S1). The combined biochemical and biophysical forces drove single-cell suspensions of FRCs into reticular 3D clusters. The tunable cross-linking density is conducive to controlling cluster size and uniformity. In all cases, avidin was added below the saturating concentration with respect to the moles of biotin in EAKbt. The clustered FRCs exhibit spherical morphologies while retaining the characteristic surface marker GP38. The porous network allows for nutrients and waste exchanges, which is conducive to retaining T cells. One notable observation is that a number of clusters formed interconnections, which is reminiscence of the tunneling nanotubes found in spheroids of MSCs, which facilitate cytosolic exchange and believed to abrogate senescence acquired in later cell passages.^33,59^

The structural integrity of the cell clusters is enhanced by avidin cross-linking, as evidenced in the shear stress test using a needle syringe injection. This is likely due to the compactness of the clusters, which is conducive to laminar flow.^60^ In general, the size of spheroids is a critical parameter in biological functions, as diameter >200 μm would create a hypoxic microenvironment and impede engraftment in vivo.^61,62^ On the other hand, cell clusters below a certain size might not provide the concentration gradients of chemokines effective for attracting T cells. From a scaffolding perspective, the high surface to volume ratio allows for exponential increase in fibril–cell interactions. We postulate that the biodegradable fibrils provide transient guides for the deposition of matrix proteins.^63−67^ It is possible that EAKbt-av initiates the budding of the clusters from the single cells seeded in the small volume. The 3D scaffolding allows FRCs harvested by using trypsinization to reconstitute their surface receptors. We speculate that the soft, malleable fibrillar networks of EAKbt-av allow for mutually supportive dynamic remodeling of the cells in close proximity. When placed in vivo, the fibrillar scaffold would be replaced by collagen and other matrix components deposited by FRCs, thus morphing into lymphoid-like tissues. The porous architecture of clFRCs allows infiltration of lymphocytes, potentially skewing their gene expressions and phenotypes. This was evidenced by the reduced proliferation of Jurkat cells and in the allogeneic PBMC cocultures, which show downregulation of GZMA, a biomarker upregulated in GVHD.^51,68,69^ Taken together, our findings support the potential of developing clFRCs as an injectable delivery formulation for human FRCs.

## 5 Conclusions

The results presented here demonstrate the feasibility of a biomaterial strategy to induce the assembly of human FRCs into 3D clusters. The formulated compact clusters containing metabolically active cells retain the attributes of lymph node stromal cells and are capable of engaging T cells. The injectable biomaterial FRC formulation should be investigated further as a platform for the 3D delivery of lymph node stromal cells.

## Supporting Information Available

Robust formation of FRC clusters, reproducibility of formation, effect of avidin, coculture with human PBMCs, congregation of immune cells around clFRCs (PDF)
