# Supplementary material for: Fibril-Guided Three-Dimensional Assembly of Human Fibroblastic Reticular Cells
Source: ACS Appl Bio Mater. 2024 May 28;7(6):3953–63. doi: 10.1021/acsabm.4c00331 (PMC11190984; doi:10.1021/acsabm.4c00331)
Supplement: Supplementary file 1 — mt4c00331_si_001.pdf [file mt4c00331_si_001.pdf]

Supporting Information

Fibril-Guided 3-Dimensional Assembly of Human Fibroblastic Reticular Cells

Authors

Ketki Y. Velankar[1,$], Wen Liu[2, $], Paul R. Hartmeier[1], Samuel R. Veleke[1], Gayathri Aparnasai Reddy[1], Benjamin Clegg[3], Ellen S. Gawalt[3,5], Yong Fan[2,4,*], and Wilson S. Meng [1,5, *]

Affiliations

[1]Graduate School of Pharmaceutical Sciences, Duquesne University, Pittsburgh, Pennsylvania 15282, USA

[2]Allegheny Health Network Cancer Institute, Allegheny Health Network, Pittsburgh, Pennsylvania 15212, USA

[3]Department of Chemistry and Biochemistry, Duquesne University, Pennsylvania 15282, USA

[4]Department of Biomedical Engineering, Carnegie Mellon University, Pennsylvania 15213, USA

[5]McGowan Institute for Regenerative Medicine, University of Pittsburgh, Pennsylvania 15213, USA

[$]KYV and WL contributed equally to this paper

[*]Corresponding authors: meng@duq.edu and yong.fan@ahn.org

Supporting information

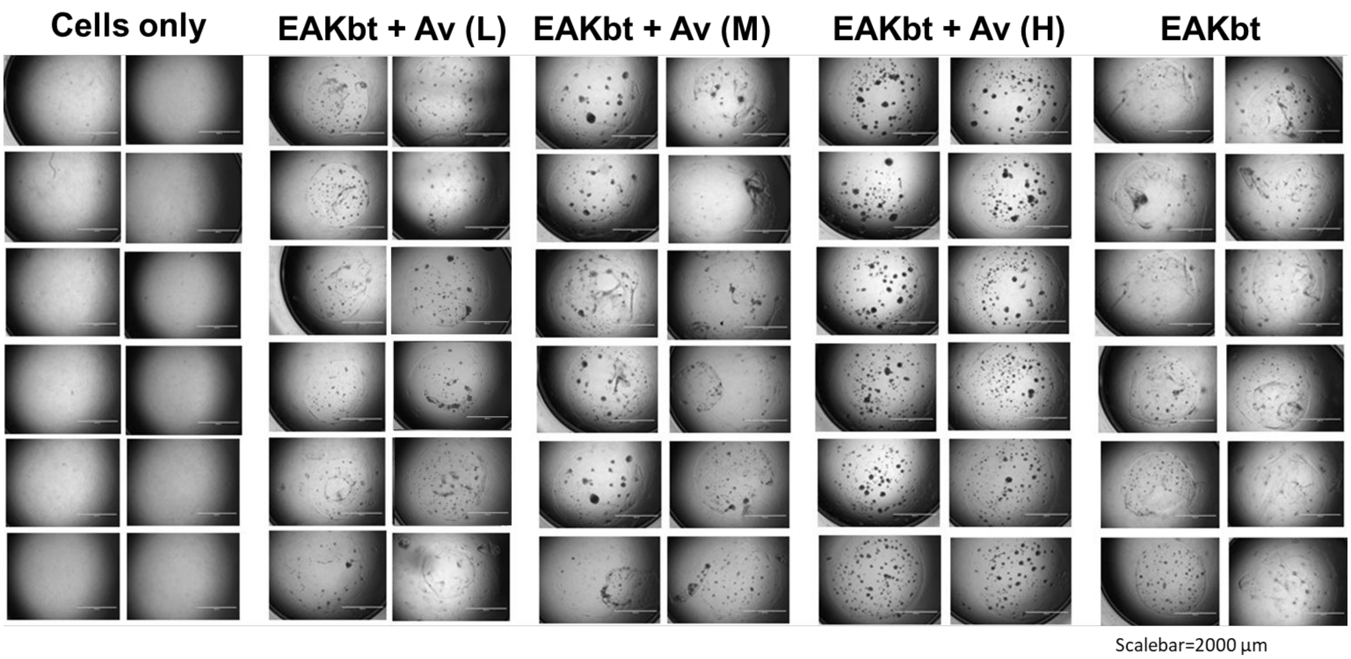

Figure S1 Generation of stable FRC clusters in EAKbt-av as a function of avidin concentration is a robust and reproducible process; BF images captures between day 1 to day 7 of FRC culture in the biomaterial.

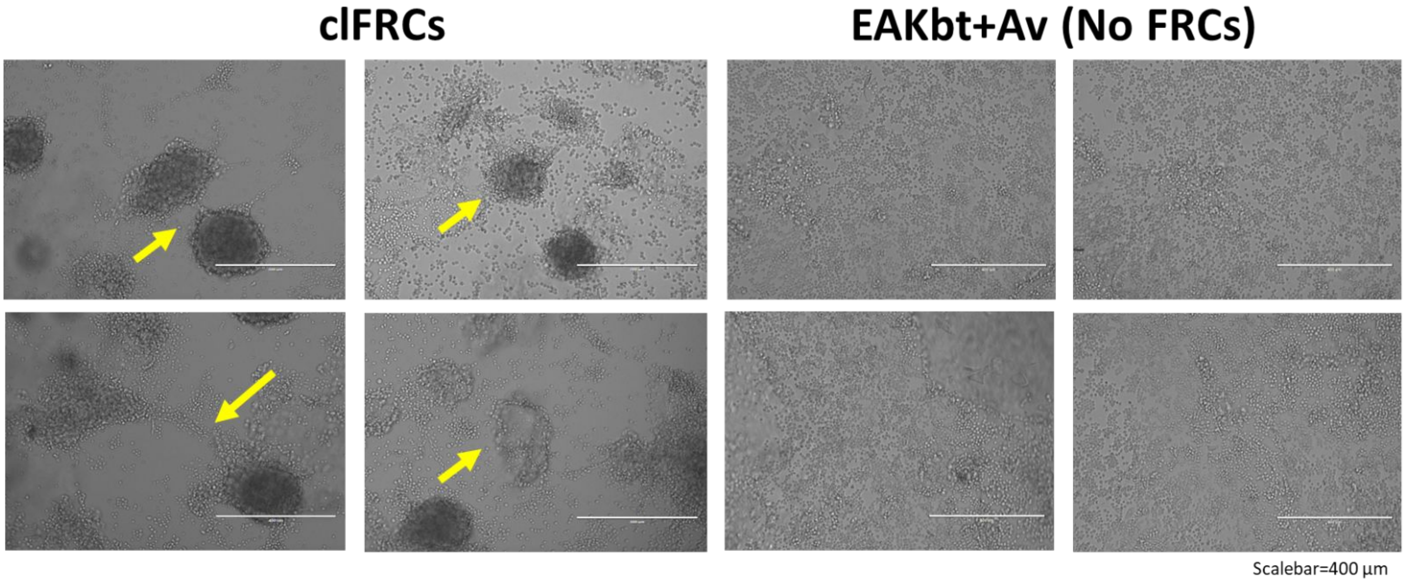

Figure S2 Co-culture of clFRC with human PBMCs. A structured assembly of CD3/CD28 activated PBMCs (small circles) was observed with co-culturing with clFRCs where yellow arrows show representative congregation of cells around FRC clusters; the EAKbt-avidin control without FRCs does not show any immune cell arrangement indicative that the congregation was not a function of the scaffold and attributed to the clFRCs.
